# Evolution of Host Specificity by Malaria Parasites through Altered Mechanisms Controlling Genome Maintenance

**DOI:** 10.1128/mBio.03272-19

**Published:** 2020-03-17

**Authors:** Michelle C. Siao, Janus Borner, Susan L. Perkins, Kirk W. Deitsch, Laura A. Kirkman

**Affiliations:** a Tri-Institutional MD-PhD Program, Weill Cornell Medical College, New York, New York, USA; b Sackler Institute for Comparative Genomics & Division of Invertebrate Zoology, American Museum of Natural History, New York, New York, USA; c Institute of Evolutionary Ecology and Conservation Genomics, University of Ulm, Ulm, Germany; d Department of Microbiology and Immunology, Weill Cornell Medical College, New York, New York, USA; e Department of Internal Medicine, Division of Infectious Diseases, Weill Cornell Medical College, New York, New York, USA; University of Geneva

**Keywords:** DNA repair, antigenic variation, evolution, malaria

## Abstract

The protozoan parasites that cause malaria infect a wide variety of vertebrate hosts, including birds, reptiles, and mammals, and the evolutionary pressures inherent to the host-parasite relationship have profoundly shaped the genomes of both host and parasite. Here, we report that these selective pressures have resulted in unexpected alterations to one of the most basic aspects of eukaryotic biology, the maintenance of genome integrity through DNA repair. Malaria parasites that infect humans continuously generate genetic diversity within their antigen-encoding gene families through frequent ectopic recombination between gene family members, a process that is a crucial feature of the persistence of malaria globally. The continuous generation of antigen diversity ensures that different parasite isolates are antigenically distinct, thus preventing extensive cross-reactive immunity and enabling parasites to maintain stable transmission within human populations. However, the molecular basis of the recombination between gene family members is not well understood. Through computational analyses of the antigen-encoding, multicopy gene families of different *Plasmodium* species, we report the unexpected observation that malaria parasites that infect rodents do not display the same degree of antigen diversity as observed in Plasmodium falciparum and appear to undergo significantly less ectopic recombination. Using comparative genomics, we also identify key molecular components of the diversification process, thus shedding new light on how malaria parasites balance the maintenance of genome integrity with the requirement for continuous genetic diversification.

## Abstract (precis)

Malaria remains one of the most prevalent and deadly infectious diseases of the developing world, causing approximately 228 million clinical cases and nearly half a million deaths annually. The disease is caused by protozoan parasites of the genus *Plasmodium*, and of the five species capable of infecting humans, infections with P. falciparum are the most severe. In addition to the parasites that infect people, there are hundreds of additional species that infect birds, reptiles, and other mammals, each exquisitely evolved to meet the specific challenges inherent to survival within their respective hosts. By comparing the unique strategies that each species has evolved, key insights into host-parasite interactions can be gained, including discoveries regarding the pathogenesis of human disease. Here, we describe the surprising observation that closely related parasites with different hosts have evolved remarkably different methods for repairing their genomes. This observation has important implications for the ability of parasites to maintain chronic infections and for the development of host immunity.

## OBSERVATION

The coevolution of host and parasite, continuously adapting to each other for survival, is described by the Red Queen hypothesis in which “it takes all the running you can do, to keep in the same place” (1). This dynamic interaction is exemplified by malaria parasites, which are thought to have exerted the strongest known selective pressure on the human genome over the last 10,000 years (2), including numerous polymorphisms of red blood cell genes (3, 4). Different species of *Plasmodium* infect a broad range of vertebrate hosts, enabling a comparative analysis of adaptations particular to each specific host. Such comparisons have revealed unexpected changes in basic aspects of cell biology, from components of transcriptional machinery (5) to chromatin modifiers (6) and lipid metabolism (7–9), providing deep insights into the evolutionary pressures shaping these parasites, including aspects important for the human disease including pathogenesis, immune evasion, and transmission dynamics.

One unanticipated adaptation of all malaria parasites is the loss of classical nonhomologous end joining (cNHEJ), a fundamental mechanism responsible for repair of DNA double-strand breaks (DSBs). Malaria parasites depend almost entirely on homologous recombination (HR) to maintain genome integrity, despite spending most of their life cycle as haploid organisms and thus lacking the homologous chromosomes typically used for repair by HR (10). The loss of cNHEJ has been described in multiple parasitic lineages with several hypotheses put forward for how this may impact genome evolution and pathogenesis (11). We recently proposed a possible selective advantage for the loss of cNHEJ in the human malaria parasite Plasmodium falciparum (12). Within their vertebrate host, parasites avoid antibody-mediated clearance by varying the antigens that they express on the red cell surface, thus greatly extending the length of infections. This process, called antigenic variation, is dependent on extensive variability within the multigene families that encode these surface antigens (13). Furthermore, to enable reinfection of a previously infected host, different parasite strains must encode different repertoires of variant antigens. Thus, the capacity to generate new variants enables persistence within a host population even when most potential host organisms have developed clinical immunity, as is observed for P. falciparum infections in humans. The primary driving force for variant gene diversification is recombination between gene copies (14). In addition to sexual recombination, recombination can also occur between nonsyntenic genes (genes in different positions of the genome; this is also called ectopic recombination) during asexual replication when the parasites are haploid (15–17). This occurs when DNA DSBs arise in multigene family members. In the absence of NHEJ, such breaks must be repaired by HR using alternative members of the family from other positions in the genome as the template for repair (15, 16, 18). Thus, recombination between genes is not limited by genomic position, and diversification is greatly accelerated, resulting in an extraordinary degree of sequence diversity (19, 20). The selective pressure to continuously derive new variants through HR could provide a selective advantage for the loss of efficient NHEJ.

**FIG 1:**
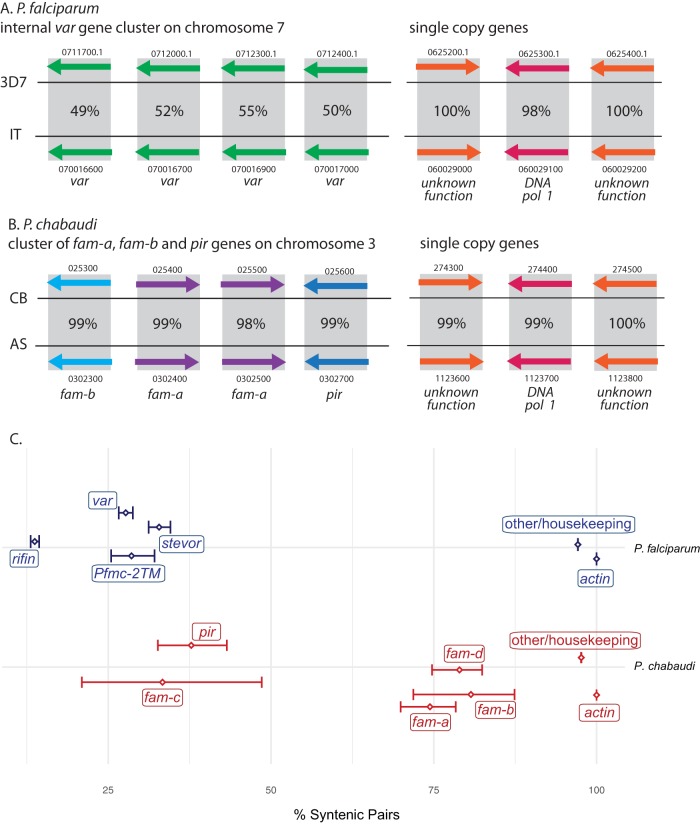
Comparison of variant antigen diversity in human and rodent malaria parasites. (A) (Left) Schematically shown are four members of the multicopy variant antigen *var* gene family of the human parasite P. falciparum. Genes from two geographical isolates (3D7 and IT) are shown from a syntenic region of chromosome 7, and the percentage of nucleotide identity between each gene is provided in the gray box enclosing each gene pair. Annotation numbers corresponding to the Eukaryotic Pathogen Genomics Database Resource (Release 45, EuPathDB, eupathdb.org [35]) are included above each arrow. (Right) A similar schematic shows the near-complete sequence identity observed for single-copy housekeeping genes. (B) A similar analysis as shown in panel A for two isolates (CB and AS) of the rodent parasite P. chabaudi. (Left) Members of the variant gene families *fam-a*, *fam-b*, and *pir*. (Right) Single-copy housekeeping genes. Sequence identities were calculated using Needleman-Wunsch alignment of two sequences (36). (C) Assessment of recombination within the multigene families. Individual genes from 15 independent isolates of P. falciparum (top, blue text) were compared to the 3D7 reference genome to identify the ortholog with the highest-scoring sequence alignment. For single-copy housekeeping genes, isolate-to-reference gene pairs were in the syntenic position of the genome nearly 100% of the time (right); such pairs for members of the *var*, *rifin*, *stevor*, and *Pfmc-2TM* variant gene families were seldom syntenic (left), indicating extensive recombination throughout these families. Similarly, 4 isolates of the rodent malaria parasite P. chabaudi (bottom, red) were compared to the AS reference genome and demonstrated that a large majority of the *fam-a*, *fam-b*, and *fam-d* multigene family members maintained synteny, even across two subspecies. Diamonds represent percent synteny with 95% confidence intervals shown by error bars. See Text S1 in the supplemental material for details of sequence analysis.

Given the importance of the parasite’s ability to shuffle sequences between multigene family members, we were interested in defining the molecular basis underlying this process. We were therefore intrigued to find that, in stark contrast to the high degree of sequence divergence observed in P. falciparum, multigene family members in specific genomic positions were often nearly identical in different isolates of the rodent parasite Plasmodium chabaudi (Fig. 1A and B). A previous study similarly found that the multigene repertoires are often conserved between these two isolates (21), suggesting that recombination events are somehow more constrained in P. chabaudi than in P. falciparum. We were therefore curious if this observation could possibly provide clues to the underlying mechanism of diversification. For a more comprehensive study of recombination within these multigene families, we expanded our analysis to examine the genome assemblies of 16 P. falciparum isolates and five isolates from two subspecies of P. chabaudi (22). The availability of long-read sequencing of these genomes enabled our comparisons of gene variability with particular attention to syntenic genes, of multicopy gene families. For each gene with a mapped position, we systematically searched for the ortholog in the reference genome (3D7 for P. falciparum and AS for P. chabaudi) with the highest-scoring alignment and then determined whether the paired sequences were located at comparable positions of their respective genomes. For single-copy housekeeping genes in both species, we found nearly universal synteny, as expected (Fig. 1C). For the multigene families *var*, *rifin*, *stevor*, and *Pfmc-2TM* of P. falciparum (23–25), gene pairs with the highest sequence identity were seldom syntenic (Fig. 1C, top), suggesting that recombination between nonsyntenic family members is common. In contrast, for the P. chabaudi isolates, the majority of *fam-a*, *fam-b*, and *fam-d* gene family members displayed the greatest sequence similarity to genes at the same genomic position (Fig. 1C, bottom), indicating relatively infrequent recombination between nonsyntenic genes. This pattern held even though the rodent parasite isolates examined represent two different subspecies of P. chabaudi. The trend was less pronounced for the *pir* gene family, which includes the *fam-c* subfamily and has been observed to display greater overall heterogeneity than the other variant gene families (26).

10.1128/mBio.03272-19.1 TEXT S1 Details of origins of sequence, sequence analysis, and link to code used. Download Text S1, PDF file, 0.03 MB. Copyright © 2020 Siao et al. 2020 Siao et al. This content is distributed under the terms of the Creative Commons Attribution 4.0 International license.

**FIG 2:**
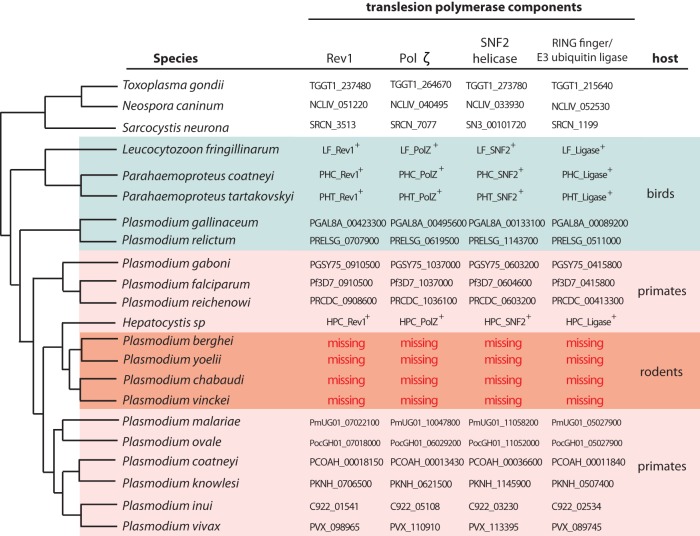
Phylogenetic tree of apicomplexan parasites based on the phylogeny of Galen et al. (37) showing the loss of translesion polymerases in different parasite lineages. Toxoplasma gondii, Neospora caninum, and Sarcocystis neurona are parasites that do not possess large multigene families and do not undergo antigenic variation. Clades of parasites that infect red blood cells are highlighted with different-colored shading according to their vertebrate hosts. Rev1, Pol ζ, an SNF2 helicase, and a RING finger/E3 ubiquitin ligase are required for translesion polymerase activity. This analysis shows the loss of translesion polymerases in parasites that infect rodents. Gene annotation numbers are provided next to each species name for all species catalogued in the EuPathDB database (Release 45, EuPathDB, eupathdb.org [35]). Additional orthologous sequences were obtained from the fragmented genome assembly of Parahaemoproteus tartakovskyi (38), the transcriptome data sets of Parahaemoproteus coatneyi and Leucocytozoon fringillinarum (39), and sequence data of a *Hepatocystis* parasite that were mined from the transcriptome of a Ugandan red colobus monkey (40) using the ContamFinder pipeline (41). ^+^, see Fig. S1 in the supplemental material for sequences and alignments of genes not previously annotated.

Given that both P. chabaudi and P. falciparum similarly lack cNHEJ and depend on HR for DNA double-strand break repair, an additional hypothesis is required to explain why members of the variant antigen gene families of rodent parasites appear to undergo significantly less extensive recombination among nonsyntenic genes. Generally among eukaryotes, translesion polymerases are required for efficient HR when the recombining sequences differ substantially (27), as they typically do when two nonidentical variant antigen genes recombine. We observed that two translesion polymerases (orthologs of Rev1 and Pol ζ) and two accessory proteins (an SNF2 helicase and a RING finger/E3 ubiquitin ligase) are encoded in the genomes of most *Plasmodium* species and several related parasites spanning a range of vertebrate hosts (Fig. 2). Remarkably, all four of these genes are missing from the genomes of rodent malaria parasites, providing a likely explanation for the reduced degree of recombination and diversification that we detected within their multigene families. This led us to question what selective pressures could have resulted in the loss of this highly conserved DNA repair pathway specifically within the rodent malaria evolutionary lineage. Although many multigene family members of rodent parasites have been presumed to encode variant surface antigens as in primate malaria species, recent reports offer a different model in which different gene family members instead evolved distinct functions (28). If at least a subset of these genes perform distinct functions, these functions could be disrupted by recombination, thus favoring a mechanism to suppress recombination between nonsyntenic gene copies, such as through the observed loss of translesion polymerases. The reduction of antigenic diversity between isolates, as we found in rodent parasites, would presumably impair reinfection of hosts that have previously harbored an infection. However, several aspects of rodent parasite infections could influence the need for continuous diversification of antigen-encoding gene families, including the length and chronicity of infections, rates of transmission and the likelihood of reinfection, and the virulence of infections as well as the average life span of the hosts and their typical number of offspring. In addition, further analysis of rodent parasite genomes could reveal potential alternative mechanisms for DNA repair that perhaps partially compensate for the loss of translesion polymerases. For example, it is not clear how the *pir/fam-c* gene family members have acquired a much higher degree of diversity than the largely conserved *fam-a*, *fam-b*, and *fam-d* gene families, particularly considering that these families consist of similar numbers of genes and are located interspersed with one another within the parasite’s genome.

10.1128/mBio.03272-19.2 FIG S1 Amino acid sequence alignments for orthologs of the four components of the translesion polymerases described in the main text. Sequences for P. falciparum were obtained from the EuPathDB database, while the additional orthologous sequences were obtained from the fragmented genome assembly of *Parahaemoproteus tartakovskyi* (S. Bensch, B. Canback, J. D. DeBarry, T. Johansson, et al., Genome Biol Evol 8:1361–1373, 2016, https://doi.org/10.1093/gbe/evw081), the transcriptome data sets of *Parahaemoproteus coatneyi* and *L*. *fringillinarum* (S. C. Galen, J. Borner, J. L. Williamson, C. C. Witt, and S. L. Perkins, Mol Ecol Resour 20:14–28, 2019, https://doi.org/10.1111/1755-0998.13091), and sequence data of a *Hepatocystis* parasite that were mined from the transcriptome of a Ugandan red colobus monkey (N. D. Simons, G. N. Eick, M. J. Ruiz-Lopez, D. Hyeroba, et al., Genome Biol Evol 11:1630–1643, 2019, https://doi.org/10.1093/gbe/evz099) using the ContamFinder pipeline (J. Borner and T. Burmester, BMC Genomics 18:100, 2017, https://doi.org/10.1186/s12864-017-3504-1). Alignments were generated using the Constraint-based Multiple Alignment Tool (COBALT; J. S. Papadopoulos and R. Agarwala, Bioinformatics 23:1073–1079, 2007, https://doi.org/10.1093/bioinformatics/btm076) available through the National Library of Medicine, National Center for Biotechnology Information website. Alignments are displayed in the “compact” format to reduce space. Unaligned regions are displayed as [X] where X denotes the number of residues for a sequence in the unaligned range. Download FIG S1, PDF file, 0.2 MB. Copyright © 2020 Siao et al. 2020 Siao et al. This content is distributed under the terms of the Creative Commons Attribution 4.0 International license.

Taken together, our observations suggest a key role for translesion polymerases in diversification of malaria parasite antigens. In model eukaryotes, these enzymes interface with nucleotide excision (29), base excision (30), and mismatch repair pathways (30), which are thought to be a major source of mutations leading to drug resistance in naturally circulating malaria parasites (31–34). Translesion polymerases may therefore play an underappreciated role in the continued threat of malaria to human health globally. This work underscores the power of comparative evolutionary studies to advance our understanding of parasite gene function and host-parasite interactions.
