# Supplementary material for: Evolution of Host Specificity by Malaria Parasites through Altered Mechanisms Controlling Genome Maintenance
Source: mBio. 2020 Mar 17;11(2):e03272-19. doi: 10.1128/mBio.03272-19 (PMC7078485; doi:10.1128/mBio.03272-19)
Supplement: TEXT S1 [file mBio.03272-19-s0001.pdf]

Methods for sequence analysis to identify synteny for gene families (Figure 1C)

This analysis is primarily based on files from <ftp://ftp.sanger.ac.uk/pub/project/pathogens/Plasmodium/ (Lin *et al.*, 2018; Otto *et al.*, 2018; Otto *et al*., 2014). Using custom code in R 3.4.0 (R Core Team, 2017) with the *tidyverse* (v.1.2.1; Wickham, 2017) and *Biostrings* (v.2.44.2; Pagès *et al*,. 2017) packages, .embl files were parsed for (spliced) coding sequence and associated information, and BLAST databases (Camacho *et al.*, 2009) were locally created from .fasta files of reference lines PF3D7 and PCHAS. For *P. chabaudi*, version 1 files were used for isolates AJ, DK, and DS (Otto *et al*., 2014); version 2 was available and therefore used for CB (Lin *et al*., 2018). Non-nuclear sequences were excluded.

For each isolate gene, the one-to-one reference orthologue was recorded when possible using the original file's annotation, breaking ties with a previous gene ID alias if needed and possible. When a unique reference orthologue was not provided in the file, the isolate gene sequence was used as a query for a local nucleotide BLAST search of the reference genome. For this and subsequent BLAST searches, the coding sequence with the highest cumulative bit score was used as a reference orthologue of comparison. For genes with still no annotated reference orthologue sequence, using the *reutils* package (version 0.2.3, Schöfl, 2016), (1) the isolate gene sequence was used as a query for a local BLAST search of the corresponding taxon-specific NCBI BLAST databases (i.e. *P. falciparum*, *P. chabaudi chabaudi*, or *P. chabaudi adami*) or (2) the isolate sequence used as a query of a remote NCBI BLAST search of the corresponding reference genome (i.e. *P. falciparum* or *P. chabaudi*).

Each isolate gene was then associated with the nearest recorded reference neighbors, i.e. the reference orthologues of the isolate genes to the left or right on the isolate chromosome. A gene was called syntenic if at least one of those reference neighbors was (1) on the same chromosome number as the isolate gene and (2) had a reference ID number (assigned sequentially on the reference genome) placing it within the same chromosomal region. Isolate genes were assigned to a multigene family, actin, or other/housekeeping based on the isolate and reference gene product annotations.

The finite population analogue (Cochran, 1977; Wendell and Schmee, 2001) of the Clopper-Pearson interval was used to generate an exact confidence interval for the rates of synteny within each gene family per isolate.

Code availability. The full reproducible code used for data analysis and presentation will be available at https://github.com/siaomc/pgfamilies.

References

Camacho C, Coulouris G, Avagyan V, Ma N, Papadopoulos J, Bealer K, Madden TL. BLAST+: architecture and applications. *BMC Bioinformatics* 2009 10:421. doi: 10.1186/1471-2105-10-421. PMID: 30542666.

Cochran WG. *Sampling Techniques* 1977, 3rd ed., New York: Wiley, 57.

Lin JW, Reid AJ, Cunningham D, Böhme U, Tumwine I, Keller-Mclaughlin S, Sanders M, Berriman M, Langhorne J. Genomic and transcriptomic comparisons of closely related malaria parasites differing in virulence and sequestration pattern. *Wellcome Open Res*. 2018, 3:142. doi: 10.12688/wellcomeopenres.14797.2. PMID: 30542666.

Otto TD, Böhme U, Jackson AP, Hunt M, Franke-Fayard B, Hoeijmakers WA, Religa AA, Robertson L, Sanders M, Ogun SA, Cunningham D, Erhart A, Billker O, Khan SM, Stunnenberg HG, Langhorne J, Holder AA, Waters AP, Newbold CI, Pain A, Berriman M, Janse CJ. A comprehensive evaluation of rodent malaria parasite genomes and gene expression. *BMC Biol.* 2014, 12:86. doi: 10.1186/s12915-014-0086-0. PMID: 25359557.

Pagès H, Aboyoun P, Gentleman R, DebRoy S. Biostrings: String objects representing biological sequences, and matching algorithms. R package version 2.44.2, 2017.

Schöfl, G. reutils, R package version 0.2.3, 2016. https://cran.r-project.org/web/packages/reutils/index.html.

Wendell JP and J Schmee. Likelihood Confidence Intervals for Proportions in Finite Populations, *Am Stat.* 2001, 55:1, 55-61. doi: 10.1198/000313001300339941.

Wickham H. tidyverse, R package version 1.2.1, 2017. https://cran.r-project.org/web/packages/tidyverse/index.html.
