# Supplementary material for: Evolution of Host Specificity by Malaria Parasites through Altered Mechanisms Controlling Genome Maintenance
Source: mBio. 2020 Mar 17;11(2):e03272-19. doi: 10.1128/mBio.03272-19 (PMC7078485; doi:10.1128/mBio.03272-19)
Supplement: FIG S1 [file mBio.03272-19-sf001.pdf]

Supplemental Figure 1:

Rev1 Alignment

```
Pf3D7_0910500     mdieyggardfekyvcnkeekiplsynkkylaeieeshkkck-ndsllfmncnFYIDDFVSFFSICNTldnsntqtkciY
LF_Rev1           --------------------------------------------------------------------------------
PHC_Rev1          mdmeyggarsmglyfrnkekrtprsykkkynveieesaknysltncriftncnFYIDDLISLSTIGNNsstntplkewaY
PHT_Rev1          --------------------------------------------------------------------------------
HPC_Rev1          -----------------------------------------------------FYIDDFVSLFPLYSS-----------Y

Pf3D7_0910500     RNekdsvedskelnnymnnnsinkttlgkfnmenkmeynytsinehnnnnyyinkihndsvfKYQDMDKTPMKNNIDNNS
LF_Rev1           --------------------------------------------------------------------------------
PHC_Rev1          PQ-----------------------------------------------------------vKRERLSESENENEKE---
PHT_Rev1          --------------------------------------------------------------KRERLSESENEKEKEKEK
HPC_Rev1          EHv---------------nntieqtp---fpyecktqikyp--neyneynkhlnstvtd-------MYRTPTKSNFYSFN

Pf3D7_0910500     NNNNNNNDnnnNNGNTYLYDNYLEDQFNQT--------------IKNRYSCTPNDQISTCTKSFNTCIKTYEEQASSSCK
LF_Rev1           ---------------------------------------------------------------------------ESPLK
PHC_Rev1          -----------YCATEVFHNTCFEDPVFVSdgsseqngvlnttpIKSECRYDEANTMYTYPSVSKTYYNTNEESAHTT-P
PHT_Rev1          ENENENENeeaYCATEVFQSNYFDDPLF--------------tpIRNECRYDETNTIYTNPSASKTCYNTNGELTYTT-S
HPC_Rev1          STLYNNDG--------MYHEYYNKSNNFMStqrsyksiidd----RNTNYIKDINGINNSYNKFDNFMNVHKNI--NSFE

Pf3D7_0910500     KNVYNSYINKKENLEILIVQNGGVIHNTLTSKVTHIISNNMALGSKKYMDYKKAIKKSKVFIVIDQYIFDCVNMQCRLPE
LF_Rev1           SITHNSYINKKERLEILIGQYGGTIHNVLTSKVTHIVSNNMSIGSKKYSDLKKKIKKWSVCIVTDSYIFDSVKEKTRLSE
PHC_Rev1          TPVHNSYINKKENLEILIVQNGGRIHNILTGEVTHIISNNMALGNKKYADFKRKVKRSKVFIIEDTYVFDCVKANSRLCE
PHT_Rev1          TPVHNSYINKKENLEILIVQNGGRIHNILTGEVTHIISNNMALGNKKYADFKRKVKRSKVFIIEDTYVFDCVKANSRLCE
HPC_Rev1          KPVQATYINKKEELEILIVQNGGILHNALTNKVKNIISNNMALGSKKYMDYKKSLKKNKIFIVVDKYIFDCVYRGKRLFE

Pf3D7_0910500     QSYLPSMLRYNCHQITEYFSLRKkdkeQNKKMQNKKIQDKINNDQNFLKEHKEGEQNnvtildtskcDKQLNDYYVEKKL
LF_Rev1           QSYLPLSLRFNSHQITEYFKLHK-----RKNNRTDHFSEKQKSLPTNIRKETERIEEfkevsrss--LAHHSTGLLQSFT
PHC_Rev1          QLYLPKTLRYNSRQITEYFPLNK----QRKRSQTNSSKSKD---------------------------------------
PHT_Rev1          QLYLPKTLRYNSRQITEFFPLNK----QRKRSQTNCSKSKDKNSENKETIEKKSVQD---------lNKDQTLNLEEDEV
HPC_Rev1          QSYL----------------INNn**------------------------------------------------------

P Pf3D7_0910500   STCHNKTSVNNNLNKTDMYGTK--edNNLLIHKDIgnnenlnyiNEQKVREENNNEEKKENITNNDN--INKHLEILNMN
LF_Rev1           RVNQSGFSNFNNIVVDTRKGNVi---HVGVPERTItyqdknplsNDNSQIHDNNNNNNNNECIENDKefIENKKKQLYLN
```

PHC_Rev1	-------------------------------EKEK---------EKEKETGMEQTEEKELNA-EENK--SSEKMKALLME
PHT_Rev1	KYCPSEGNNGNNLKNGQKEANErneeQEQEMEKEQ---------EQEKKK-KEQTEENDPSTTEEKE--SNEKMKTLLME
HPC_Rev1	---VNILNMN

Pf3D7_0910500	MDYEHVKKFIICNSNEYFKRLQEYYLEKELKENIYSNvSSNLYLNKNTFEDFCRKYRILNYLSDEEIKWLKCKSELNINI
LF_Rev1	MSYEKLITLIKTPGDEYFKMLFQKCRFMELENPVFPI-KSEVYLNGTNFEEFLKKYNIINYFDSEEIRWIREESRLNLKI
PHC_Rev1	MDYDELKKYIITDYSSYFSRLYKYYCDKERKETIFCT-TSDLYLNKNNFEEFARKYRILNYLTEEEIRWIKNKSKLNLKI
PHT_Rev1	MSYDELKKYIITNYSNYFSRLYKYYCDKERKETIFCT-TSDLYLNKNNFEEFARKHRILNYLTEEEISWIKNKSKLNLKI
HPC_Rev1	MSYINVKKYILSNSSEYFKKLYKYYIDKELKQNSFPNiQSDLFINKDTCEPFLQKYKIRNYFNMNEIKWLKNSSKINIHI

Pf3D7_0910500	KNLWENDIIHFFFDLVLRKKEHFKNNTVGnnnignnhnignnnnignnHNIGNNNNNIGNNNnntqsyTDINKsSSYICE
LF_Rev1	KNIWENDIMHFLFKMILRKYPRFSEETRK--------------------DKEKKKPSVTEEKtthny--------DSMSE
PHC_Rev1	RNIWENNFVHFFFNMVLKKK----------------------------FDVSKQNCSMTNDD------TKKDDeDEEMSD
PHT_Rev1	RNIWENDFIHFFFNMVLKKK----------------------------FDVSKQNCSMENND------TKEDG-DEEMSD
HPC_Rev1	KNVWENDMVHFFFDTVLKSYMQQNYDEKLks------------------DRGKLSEEIKETS----------------SN

Pf3D7_0910500	NLIESISAYLYNSRLYILGNWNYISKELFNFDDIKEYEKgsKKFVYLYIDFDNYFLNASIR-------nkicmYEKKKSM
LF_Rev1	GLIQHISEYLYNSRLYILGNWNYIAKLFFPFRDLREKDK--RKHVYLYIDFDYYFLNSSVNKDSYH--------YKECNM
PHC_Rev1	KLIDSISGYFNNSRLYILGNWSYITKALFDFNDIQLTAE--KKCVYLYIDYDYYFLNASINKNNND----------NKNI
PHT_Rev1	KLIDSISGYFNNSRLYILGNWSYITKALFDFNDIQLTTE--KKCAYLYIDFDYYFLNASINKNNNDkvnsfpiSYGDKNI
HPC_Rev1	ELIKTIKDYLYNSRLYVLGNWSYINKIFFNFHDIKTSKS-pNKCVYLYIDFDYYFLNASIKRGNNL-------FRSNDSF

Pf3D7_0910500	NNEIFCVCHSLKKEDSYGIISSTNYWGKKNKILKGMVKGEATK-MNKNINFVKYDFSNILKCSYLFLLVLINYSKNVRVL
LF_Rev1	NNSVLCVCHSVKKEESYANISSVNYWAQKNKIYKGMLKKEATN-LHKDIHFVKYNFSNILRNSFLFLLVLLNFSYYVKVS
PHC_Rev1	NNSIFCVCHSTKKEDSFGMISSINYWAAKNKIYKGMLKMDATRILHKTIHFIRYDFSNILKCSFLMLLVLMNYSTNVKVS
PHT_Rev1	NNSIFCVCHSTKKEDSFGMISSINYWAAKNKIYKGMLKMDATRILHKTIHFIRYDFSNILKCSFLMLLVLMNYSKNIKVS
HPC_Rev1	NNRIVCVCHSLKKENSYGIISATNYWGKKNKIIKGMVKSDVTKVHKNNIEFVKYDFSNILKCSYLFLLILINYSKYVRVI

Pf3D7_0910500	SVDESILQLFYEKEEDIFIISKKISDDIYRLTNLSVSIGISHDLSTSRKALKFCKKRFMFFDFYHHFNIFIK-KYISLKN
LF_Rev1	SIDECVLQLFYEKEEEVIQIAKRISNHISYLTDLNVTIGIAKDLASARVAAKFCKKRYMFFDFYHHFCIFLR-KYIDKQN
PHC_Rev1	SVDESVVQLFYEKKEEIFTIAKKISNDIYNLTRLNVTIGISDNLEVARHAAKFCKKRFLFFDFYHHFCIFLR-KYIDWKR
PHT_Rev1	SVDESVVQLFYTKEEEIFTIAKQISNDIYNLTRLNVTIGISDKLEVARHAVKFCKKRFLFFDFYHHFCIFLR-KYIDWKK
HPC_Rev1	SIDECILQLFYEKETDINIIANKICDDIYSLTNLVVSIGISDDLILAKHALKYCKKRYIFFDFYHHFKIFIRrKCVRGSG

Pf3D7_0910500	KES-------------NITPEASNDDNIKSNTISDDKIINGKLKHMIEQNKNVNGTNNTDDYRTNWMSDDKLKDLFKEYL
LF_Rev1	ESKM--------------------------NSMKEDFKKDLTT------TMTTTTTTTTTTTITNSSEPPDPCILFNAYL
PHC_Rev1	EGEFvdfniyylnhykNNRNKINNNNNNNNHNKKNDNRNDKNAFFMNGENK-----------------------------

```
PHT_Rev1          ER----------mqqkrKKRNEKQKQKNEVTDETKNETKNETKDITKD-ETKNETKNETKDE--TTNETKNETTDLFDVYV
HPC_Rev1          ESTS-------------------------------------------SNSCNGNSCNSNIIYELNSSEIN-VEQLFNEYM

Pf3D7_0910500     SFEKKRKSELKYNENNKEKYNMNIDNIYFMNLIK-GKREkEVEYIYNKFLEMNKEN--IEQMVNTYFEEVI-HPVSTYFF
LF_Rev1           S---EMNEETKWNTCTNEDRDRD-----FLNHVK-RIDP-EVQTLFASFMKTHKNE--LESILNTFFEKVV-HPISKHFF
PHC_Rev1          --------------------------------------------------------------------------------
PHT_Rev1          KFEEKKRKR-DFGEDEKEETQAEYIDVAFLKHVK-QKDE-EVENVFEKFMNLHQNN--LETILNTFFDKIV-HPVSKYFF
HPC_Rev1          LFEEKRGHSSINPNGTPNNMPNNTPNKYFLNGIEdKKEEkEIEILFDKFIKIHKEKkkMENILNTYYEDVTpQQVSKYFY

Pf3D7_0910500     FYKPNE-YYFNILKNMNYLNG-YFISFNMYYLPHIENDSNNNNNNNNHHHSNNNLHSNNNFHSNNNWLN--nkMDRNINK
LF_Rev1           FFKKDDaFYFKILQQFNYMHG-KFISFNIYRMFDDIEIMNEKKNTSKVPAQHSN--------------------------
PHC_Rev1          -------------------------------------------------------------------------MEVGTNP
PHT_Rev1          FYLKNN-FYFDILKKYNYMKG-KFVDFNIYYLNNESYINNNGSNNGSNNNNNNNNRKNKNKNKNTIFLNgenkMESNQNT
HPC_Rev1          FYKKNY-YHLNILNKLNYMDGgTFINLNIYY-------------NNQNDNQDDNIYANTYVDKDTKVTM-----------

Pf3D7_0910500     KELENNDEKKSINISVNYGVRFNKINDFYYLIYFMTKQLYLRLKIKNLKAKLLHVNFFIKAENENVNPMKYLGRGRVIRI
LF_Rev1           ------DLSKSINIRVNYGVRFTKINDFYFLIYFMCKQLFLRLKIKKLKARSLHISFFIKLEEAPVNPIKFLGRGKVSRA
PHC_Rev1          NISENLNETKSMSIRANYGIRFNKINDFYYLIYFMTKQLYLRLKLKNSKAKNLTMNFFLREENENVDPAKYLGRGKVINL
PHT_Rev1          GINENLNETKSMSIRANYGIRFNKINDFYYLIYFMTKQLYLRLKIKNSKAKNLSMNFFLREENENVDPAKYLGRGKVINL
HPC_Rev1          RTQNEGNEKKSINISVNYGVRFSNINDFYFLIYFMTKQLYIRLKIKKWKTKCLSVFFFIREKNENINPTKFLGRGKVYRV

Pf3D7_0910500     SSKIKLNQHTNCFFVYFFKVIHTFDFLANNLSDLRGVQIICSDTINENKTHV-NKKSILYYFyVNtekniknkknckyva
LF_Rev1           YDKVQLNHYTDSMFVFFFKTISTFRIIEDKLHELRGVELSISDIISGEINYS-NKKNILYYF------------------
PHC_Rev1          KSQIKLNNHTNFFFVYFFKVIFKFQYIMHKLEDLRGVQLTCFDIIKE-VSRP-NNKSILHYF-QN---------------
PHT_Rev1          KSQIKLNNHTNFFFVYFFKVIFKFQYIMHKLEDLRGVQLTCFDIIKE-VSHP-NNKTILHYF-ANtvkrkntstiitmsk
HPC_Rev1          GSKIKLNSYTNCFFIYFFKIIYEFSALSSKLEDLRGVQIVCSDMIHDETYYPvGKKSILHYF------------------

Pf3D7_0910500     swkddnflevpqkvndkdqvqenvkdphqinenkiHLHNIMLSSQKKNETNKKETNGKISKNNinsssilsyikdknkgt
LF_Rev1           -----------------------------------HSMNKRSIISKSKEGKEVSVDPKEKMNRvet--------------
PHC_Rev1          --------------------------------------------------------------------------------
PHT_Rev1          nskndtlslsylndstqqkmennyiketntneelvHWEGKEKEKEKGKEEGKKEKGKENEKER------------kkkll
HPC_Rev1          --------------------------------------------------------------------------------

Pf3D7_0910500     niltsqinwthSNNNNNNNNNNTYSRKEIRKTVRKGTSTSIKRKIKNKINthiryDK-------NIRNNKLYH--yeYKQ
LF_Rev1           -----------HEEETTSNNKQEEEERQITSTNKGSKDKTFKNRMTEK-------KQSNT--kkNVKKRKNEVi---KCK
PHC_Rev1          --------------------------------------------------------------------------------
PHT_Rev1          nkkpknsnnrkHNSSCNSNEGESKIKRKKRKTGIIKNEETYEKKITDYIEgfvtlKSSSTynstNVRKKKNTNdicdNTK
```

```
HPC_Rev1          ---------------------RASADKSINKSINKSINTSINKSINKSIN-----KSINT-----------------NTS

Pf3D7_0910500     NILNYFVHNNHNSNRTQNLIHKKENNFQNNQNVSLCSsskTINKGFKRKCNILRSNANfLNIRKKIKITKNYKISDFFPS
LF_Rev1           NILSYFVKN-PDIKNNNSILQISNKLIRKNNHSVLQG---YIRISSKQNQMPLKKKSS-----SRIHVYRNYRIYDFFPL
PHC_Rev1          --------------------------------------------------------------------------------
PHT_Rev1          DILSYFIKNINEVQTKNQLINRTTKIIKGTKKTQNRNncfHPLLSLTQNIKFIRSRSMhIEKYNQRKETVELEHINMFDS
HPC_Rev1          NIKKVYKKREKKQSTNSNKLSVNKKMYVNN-----------------------------LKKIAKINVTQNYKILHFFPN

Pf3D7_0910500     ILKKKIY---IRNNKNIYDDEIKTYGNGNIIMHTNIFDSIINKKIKKENdeendeene-eneqnekneknelcdknyHMK
LF_Rev1           FCKNKINretTNNESRSMDDEDDNDSSCTSDHHKNIFDSISNVKIKKEQ----------------------------RIK
PHC_Rev1          --------------------------------------------------------------------------------
PHT_Rev1          IINMKVN---VKTELDAYIEAEVSKEKEKGKGKGTEKEIEVKVQVKTEAgsgteaeteteieaeaeteikeeaeteiEKN
HPC_Rev1          ILIKRQT-----NDPKNGTKNGTKNGTKNNKVKTNMYDSIFNLKIKNEK----------------------------EML

Pf3D7_0910500     KQFTCCYFRYKEITQYL---------------VHKEKLFEENHK-----CNDLCRNDIFCFTYICNNLFLFNHTND-KLN
LF_Rev1           EEPTCCYLSYQRIHHHI---------------QVLITLE----K-----WEDDDTKNLFCYNSLCNELLLMNQHNVNQMH
PHC_Rev1          --MTCCYKFCIKIYSDV---------------KEKQNAHTHNVN-----YFVECAHHSICYICICNRLYVISQKNEEKFH
PHT_Rev1          QKMTCCYKFCITIYSDV---------------KEKQNAHTQNVN-----YFMECAHHSLCYICICNRLYTINQKNEEHFH
HPC_Rev1          EEVNNCYLCYQQICNSAtgtgtgccndigivsKIDSNATTEKISdstsvCKQNNSNGLFCYACICKDLHQFNKKND-ILS

Pf3D7_0910500     LYLYIKKIINSYRSYFHNTFEEHDCVCNNTlkkdqeNIYYLNKFMVKVIDDICEMLHKKRYIDVLQNFLKNFKYVWSLNN
LF_Rev1           LYLHIKRMILSYISYFNHLYEKHDCENTNK------DTKFVYKFVLDHINNISNTLHNYKQIDMLFTFLIHFKRVWEIYK
PHC_Rev1          FYLYVKKIFNSYISHFINILKNHKCDTNDD------DNEYLYKFLLYILNDLCEILHKKRFIDVLQKLLINFKCFWKLQK
PHT_Rev1          FYLYVKKIFNCYISHFMNILESHKCDTNNDisddvnKDKYLYKFLLYILNDLCEILHKKRFIDVLQKLLINFKCFWKLQK
HPC_Rev1          LYIYIKKIIYAYINYFNNIFCDNKCNNNCD------SQYYLQKFICYILDNICNELHDKRLIDILHIFLTNFKHVWCLDK

Pf3D7_0910500     -ELFPEIFQRILIKYninhmtt
LF_Rev1           -FPYSQILQKILIK--------
PHC_Rev1          kDLFSNLLQNILNKY-------
PHT_Rev1          kDLFSNLLQNILNKY-------
HPC_Rev1          -KPFPSIFQTIFIKYnv-----
```

DNA Polymerase ζ Alignment

```
Pf3D7_1037000     MNLAEKPKAFFICKFLFFYYIYKKPILPFDSLKCKISGKEIPYVCIIQILGLSLLGQRVCLYIHDFYPFFYLLIPPEEKQ
LF_PolZ           ------------------------------------------------------YGQQVCLYVHQFFPFFYVLIPPEEKE
PHC_PolZ          MNSLEKANVFFICKFLFFDYLMTKPKYPLDPSKCTISNRELPFVSVIRILGVTPYGQTVCLYVHDFFPFFYVLIPPEEEN
PHT_PolZ          ------------------------EKY-----------------------------------------------------
HPC_PolZ          MNIIDNPEAFFVCKFLFFYYIYKKPTLPFDSWVCKISNKKIPYVCVIQILGLTLYGQSVCMYIHGFYPFFYVLIPPEEKG

Pf3D7_1037000     NDKLEIELCSFLEEEYGKARKDPSNNV-CVYNIERVKRKGIYGYNEECDDFLKISFLYPNTINYFASLLKKKLFKKRIWD
LF_PolZ           NYDLEKELCFFLDNEYHKLGTDKNKNV-CVYNIKRVKRKCIYGYTEDYEDFLKISLLNPDVVKYVASLLKKKLFKERSWD
PHC_PolZ          NPHLERELCDFLETQSKNNKKQEGRVS-CIYKIERVKKKCIYGYTEKLDTFLKVYVLNPDWIKYLATLLKRKLFKSKSWN
PHT_PolZ          -------------------EKHEEVVS-CVYKIERVKKRCIYGYTEKWDTFLKVYVLNPNWIKYFATLLKRKLFKNKSWN
HPC_PolZ          NKNLEIEICKFLEDEYGKLKKNTNENSvCIYNIERVKRRCIYGYKECHDDFLKIYCLYPDTIYYLASYLSKPLFKNRI--

Pf3D7_1037000     LYEVHINYMLHFLCMKNIYGCSEIYIDKNIYFRKEFVNEINFECIEKEERWYLgEKKYNLD   -NFKRNKYLEVDAPLV
LF_PolZ           LYEVHTNYMLQFLCSKEICGCSDIFIGRGIQFRPDLPKDITFHQFSKVEKWDL-TLKNDFE   KKIKGSKYIEKDAPKY
PHC_PolZ          LYEVHITYMLHFLCSKNIYGGAEIYIDHNIFFRKEFPEDTLFDNFIKHEKWKV-QKRWEFD   EHIQRSKYVDKEAPLF
PHT_PolZ          LYEVHITYMLHFLCSKNIYGGAEIYIDHNVYFRKEFPEDSLFDNFIKHEKWKV-QKRWEFD   EHIQRSKYVDKRAPLF
HPC_PolZ          LYEVHINYVLHFLCSKHIYGCSKIYVNKNIFFRKDFVSSINFENFIKEDKWDV-KKKKTNT[5]ESVKKKNYISADAPYV

Pf3D7_1037000     FTIKTINVNFSSLKRETTYDIECDIKHQHILNEKIYTYEFEKNKIKWKKEFNFDLPINHLDSFAKMWMKEKKRCKYMDMD
LF_PolZ           WTYETRDVSFSTLQKETKYDIECDILHNEIMNEAICIHKFEKNKKKWKEELGQEMPLQYIETFVDIWIKEKKRCQKKYPH
PHC_PolZ          FTKKTKNVNFSKWERETTYDIECDIKHQSILNEQVYSYEFEKNKEEWKREMGFDLPVGYLDAFANLWIKEKDRCKNLNCK
PHT_PolZ          FTKKTKNINFSKWERETTYDIECDIKHQSILNEQVYSYEFEKNKKEWKREMGFDLPVNYLDAFANLWIKEKERCKEVNYK
HPC_PolZ          FTQETRNVIFSNLKKETSYDIECDILHDNILNQQIYTIKFNKYKKKWKEELNFDLPINYIDSFAKMWMKEKKRCKNVNPE

Pf3D7_1037000     LKRELFYFKSYENDLCFDTFDVLTERTKKMFQGFLEFMKRM----QSEKDKVNYEQILEYDNIENVKKYNKVINDDMKLK
LF_PolZ           LAKELFHFD-----------------------------------------------------------------------
PHC_PolZ          QLKEIFHFDKYETEVDFNTFDILTARTRRMLDDFAKFLENR-rnEKREKGNSMEPQHEAKHEAKHEAKQKGAENETKSEK
PHT_PolZ          QLKEIFYFDKYETEVDFNTFDVLTERTRRMLDDFVNFLKERqrnENRQK----ESLKETKQKEKQQEEENVTENKTETET
HPC_PolZ          LMKTIFNFDNFEKKLDFNSFDILTGRTKWLYNQLVTFIKSR---EQKNEPNIGNEKKNLSSNGKVDEKDIKTGQDMLKKG

Pf3D7_1037000     EDHNFNSDMNNIEVNIKQINNNDNINNCKNV-VDNEEENDMLSNMISNKKKDHMGISPNLF---IQNKEQEKEKQDEREK
LF_PolZ           --------------------------------------------------------------------------------
PHC_PolZ          ENKTNNETEHKPGNETKTET--------KSIKKDDETSPLSHANVIINDIILLAGPMES---KQINNKEFQKDYQMEKEK
PHT_PolZ          VNKTNNKTENKTENEIETETENKTKNEIKQIETDNKKSSLSDTDVIID-----YNPTDSIKkKQIKNKKYHNDYKVEKEK
HPC_PolZ          EIYDKKCSHSEINNNIKKSQNIEKEHNVGLNNTNNKLANTNNELANTNKEIVVMTDYKKIR-NKVNNKKVKGMASFEINK
```

```
Pf3D7_1037000        EKQDEREKEKQDEREKEKQDEREKEKQEEREKEKQEEREKEKQEEREKEKQEERECFIEYVNEKGCIASFEINKKRKDVV
LF_PolZ              --------------------------------------------------------------------------------
PHC_PolZ             IEKDYLSKKAHTDVVENHVNDSINEKKKNSVEAPCHINTKGKITGTDVKTLTNINRKANEANNCYTTKVLQSEKEGEANL
PHT_PolZ             NEKYSLSKKEHKNVVAIYSNDSENDRKENSVETTCHINTNGKITGTNVSTLTNINRRINEENNYCRSRLLQSEKEGKSYL
HPC_PolZ             KKKYVI-RYVYKKKMPIIKKGYAVSRFWRGAHNIVGSNDDAGINEDNVNGNVN-NSWNNNVNNSGNDNV---NNSGNDSV

Pf3D7_1037000        RYS[6]RINKCYAVINNLVDHISGRDNKN-   MKEKMKG---NIYNRIHDNIED    ENKEDTSKFEYIgkeNHMENK
LF_PolZ              ---   ------------------------   ------------YSK-------    ---------------------
PHC_PolZ             KNS   KNIRYTTVISKSITNEKRNNNRFI[4]LHNKLKGivrYIYKKKPPRILN[ 9]RDKTKEKEAEAEadiDKQEKE
PHT_PolZ             KNN   KQIHNSIVINKSITDEKKKNNRFI[4]LHNKLKGivrYIYKKKPPRVLN[10]KNKTKEQKPERD---DKQEKK
HPC_PolZ             NNS[1]-NDNINNSGNDNINNSGNDNNRMY   -HNQTKC---------------    -NKLEENKVR-----NKKENK

Pf3D7_1037000        ENIRKQYEQIKSDNMKKKINIKYGNIFFLEILTEIKDENCYSSDYNQDKIKAVFYIVREERLMNLYEDYNNCMGIIATKP
LF_PolZ              --------------------------------------------------------------------------------
PHC_PolZ             KCTTQKVEKTMEKILPRKINIKYGNLFFIDILTEIEGEEWYYPDALQDEIKAVFYVIEDERIKSYYENYYDVIGIIASKP
PHT_PolZ             KYSTEEVEKTMKKIVPRKINIKYGNLLFIEILTEIEGEEWYYPDALQDEIKALFYIIEDERIKSYYDNYYDVIGIIASKP
HPC_PolZ             K------------------SIKYGNIFFCEILTAIKNENCYSSNYYEDETKAIFYLIKDERILNSYEEYTNCVGIIATKP

Pf3D7_1037000        FP[75]KNVINKKNDDTYGSS[20]NNDIKKVK-RTFFDFNINYNNVNICIVENERELIQKLINKILFYSPLSIVSYEND
LF_PolZ              --    ---------------    ---------------------VNVCIVEDEIELIQKLVEKINFFCPLSIISFENH
PHC_PolZ             FN    RNIMNGLKWSQLGTK    GKDVKEKKsACSSSFLPDVDTVNICIVENEKELIMKFIELIRLFCPISIISYEND
PHT_PolZ             FN    RNVLNVLKRSQLGTK    GKDVKGNKsRNSSSFLPDVDRVNICIVENEKELIMKFIELVRLFCPVSIISYEND
HPC_PolZ             FP[ 6]---------------    ------------------------------KELYKELINKINFYCPLSIVSYEND

Pf3D7_1037000        KYNINYINQRCLALDIGNFYKMIC---KLNDQKKFLDLHNAYSRNIKGIIIESLYKLSNTYNTSFENLCKHYLNINIPSI
PF_PolZ              KYGINYINERCILLDLGNFYKLIS---KVKDQCNFLQYNNSYNKSIKGRVI---CRLADVSNSSFENICLEYLRVTIPSI
PHC_PolZ             KFSLNYINKRCTILKIGNLYKLMS---SVNNQPVFTDMNNSYNKRIKGRVIESVYRLSDESNTSFENLCKVYLNVTIPSI
PHT_PolZ             KFNLNYINKRCATLKIGNLYKLMS---NVNNQTAFTDINNSYNKRIKGRIIESVYRMSDESNTSFENLCKVYLNVTIPSI
HPC_PolZ             KYNISYINKRCLFLNIGSFYMHIStnnKLNDQYNFINLKNTHKTNIKGKIIESLYKLTNISNTSFENLCKHYLHINIPTI

Pf3D7_1037000        NKYTLYYWYNYNTKRKIKANGSSTVVNKD[7]NDMDEEHIYFPYRHITIKHYLRKVYFILLIYDKICFLKRKMNFCKYIH
LF_PolZ              SKYTLYYWYSYENKKKEDG-----LISRD   SCSKESSTFFPYRYLTMKHILMRLNLIQLVYEKINFLKRKMSFAKYLH
PHC_PolZ             SKHMLYYWYIYSSG-YKDNRHGTCIDSDN   SCKDNNCIYFPYRYIVIKHMLMRVIFTKQIYEKINFFKKKIGFAKYTH
PHT_PolZ             SKHLLYYWYIYKKDMYKDTRCVPFIDNDN   NCGNNSCMYFPYRDIVIKHMLMRIIFTKQIYEKINFFKKKIGFAKYTH
HPC_PolZ             SKYTLYSWYMYNEQ-------------DN   ------------------------------------------------

Pf3D7_1037000        VDLLSLINRGSQYIIESFLLKMSIKYNYVLYSPSNKEIFDQRPILHTPLILQPKSSINFFPLLVFDFQSLYPSILIAFNI
```

```
LF_PolZ          VDFLSLINRGSQYIIESFLLKLAMRNDYLLFSPSNKEIFHQRPIMYTPLILQPLSSINFFPLLVFEFQSLYASIAIAFNI
PHC_PolZ         VDILSLITRGSQFIIESFLLKISLKRNFLLYSPSIQEISNQRPILHTPLVLQPQSSFQFFPLLVFDFQSLYASILIAFNI
PHT_PolZ         VDILSLITRGSQFIIESFLLKISLKRNFLLYSPSIQEISNQRPILHTPLVLQPQSSFQFFPLLVFDFQSLYASILIAFNI
HPC_PolZ         --------------------------------------------------------------------------------

Pf3D7_1037000    CYSTCLGTITLKR[13]KDENIEGSLMEKCETYKNGINDKNhiyDKNELSCSNKNDLPSDEYTDMMNISEDAKYLIDLLD
LF_PolZ          CYTTCIGTLTKKR    KTQVEKIHQEEEEKEEHTNINISN---DSKNMNPINMNNTTNTALQLNFEEKKNIEYEFNDLN
PHC_PolZ         CYTTCIGTLCRKS[13]SSKNERIPLRESVEKTDKPLQTNQ---DKFQTNKSKTKWRVFDLEIYQIYENKQSSVRYVSSS
PHT_PolZ         CYTTCIGTLCRK-    --KNERIPLRESVEQTDKTIQTDH---DKFQTNKSKTKWRVFDLETFQMYENKQSSIHYVSTS
HPC_PolZ         -------------    ----------------------------------------------------------------

Pf3D7_1037000    ENIND[21]GENIPSENMITED[4]KYKLDMNNKLEMGN[14]NIN[7]EEKSLESNFEFIKLGVMKNVPSISSRIKNLK
LF_PolZ          EKHNE    DEEEEEEEEEQEN[4]IHANHLNDDSQSKN    NGE   ETSEEEIIYDFLKLGVKKKDPRIMRRVKQLN
PHC_PolZ         EKKKE    GEDIRREEYSGNE   IFENEKNDKKKI--    ---   --SDEEIPFEFIKLGVKGKDETVMEKVKDFR
PHT_PolZ         EKKKE    GENTRREDYRNNG   IFENEKNKDKEMVL    NEE[7]TKSDKNIPFEFIKLGVKEKDETVMEKVKYFQ
HPC_PolZ         -----    -------------   --------------    ---   --------FEFIRLGVKKSAPDMRNTLKNIK

Pf3D7_1037000    SEDIIITSNNTIYVKKHKRKGICPLFLEDILKTRIMLKRCMGMYEER-VNKKLNERMGKLKLILNVATGYIGANFSGRMP
LF_PolZ          RNDLIITSNNTIYVTREKRKGLFSIFLEDILKSRIMLKRCIEKYNEPRIKKKLEKRVHLFKLVLNVAIGYIGASFSGRMP
PHC_PolZ         KDDMVITSNNTVYVSRKIRKGICAVFLEDILKTRIMLKRCMNIHSDKYIKKKLSDRTFIFKLILNVAVGYIGASFSGRMP
PHT_PolZ         KEDIIITSNNTIYVSRKIRKGICSVFLEDILRTRIMLKRCVNIHSDKYIKKKLSDRTFIFKLILNVAVGYIGANFSGRMP
HPC_PolZ         SDDFIISSNNTVYVKKEKRKGICVCFLEDILKTRIMLKNCIVICNGR--ETQLKEKIQKLKMILNTAAGYIGASFSGRMP

Pf3D7_1037000    CVDISESIISIGRNFLLFIIEYIKENYKFVKILYGDTDSLFLLNETtDDIQSSFKLAYEILNSINNILPLPMYLNFEKIY
LF_PolZ          CLDISESVISLAKNSLMFIIQYIKNNYANTQILYGDTDSLFLLSKC-DDMLYSFCLAHHITTHINNIVPTPMCLIFEKIY
PHC_PolZ         CVDISESVISIGKNSLAFIIEYIKRKYINVDVLYGDTDSLFLLNKT-DNLHYSFQLAYNIINTINSILPPPMFLNFEKIY
PHT_PolZ         CVDISESVISIGKNSLAFIIEYIKRKYTNVDVLYGDTDSLFLLNKT-DNLHYSFQLAYNIINTINSILPPPMFLNFEKIY
HPC_PolZ         CVDISESIICTGKNFLLFLIEYIRINYKYIKILYGDTDSLFLLNEI-DDLKYSFNIAYDILNKINNILPKPMFLNFEKIY

Pf3D7_1037000    CPSLLLTKKRYFGFSFKNENQDNPILDLKGVESIRSDQCILVKNILIQIYFILFYFKNNCYFSYCCCCCYLCKNFFSNLI
LF_PolZ          SPSFLLTKKKYFGFAYKNEKIKKPILEIKGVESIRSDQCELVKKILKQVYFIFYYFKNHSYISRCCCCCYLCSRFFNNFI
PHC_PolZ         TPSLLLTKKRYFGFCYKNEFQKKPVLEIKGIESMRSDQCDLVKKILIQIYFVFFYFKKNSYISSCCCCCSLCSNFFSNLF
PHT_PolZ         TPSLLLTKKRYFGFCYKNEFQKKPVLEIKGIESMRSDQCDLVKKILIQIYFVFFYFKKNSYISSCCCCCFLCSNFFSNLF
HPC_PolZ         CPSVLLTKKRYFGFAYKTENVKKGQIEIKGLESIRSDQCELVKNILKQIYFIFYYFKNTCYFSYCCSCCYMCVNFFTNFV

Pf3D7_1037000    CSC--KELNVHKTDPCFLFTLLKIVLSicKVRRYINQNEDKYNinNNNNNNN[31]DKVKLYKEVMNWVEYYDNENLLLK
LF_PolZ          CNC--EKTGIHQKDHCFLSELLSTI-----LEIPLNDKLDKTElpKEGNTEK    ERRYYYDELLHLLNF-DTHNLRLQ
```

```
PHC_PolZ             CSCtcKESKTPPNRPCFLSKLVKVVYP--ELQEFENNSLQKTT--KENWTKE    TKKQFFDKCLQLLTKKEGNNEILK
PHT_PolZ             CSCtcKKSKTFPNRPCLLSKLLKVLFP--ELEEFTNNKLQKMT--KTNETNE    TKKQFFEKCLQLLTKKEGNNDILK
HPC_PolZ             LYC--NVSYIHRKEPCDKLKLYKDIIK-----------LIKMD--------K    ------------------NNTVLK

Pf3D7_1037000        TLNFLYREKYSFLIYLFNLFDEATLHKEIEKIIQTNFEKIQNQKNN-KCCFINPSNVFKSCLCVKNI-NENIKCDQIRCY
LF_PolZ              MLHYLYNEKYSYLMYTYTYHGPGILKEKVKKIIEKDFDKLCFSAHN-NCCLVDPNNHFNICLCVKEE-NRNLKCSKNTCY
PHC_PolZ             TLNFLYKEKYNHLFYIFTHFDSVTFKREVGNLIKKHKDEIVLDRYDyDCCFNSEGNVFNLCFCINEK-DRKYKCDNTKCY
PHT_PolZ             ILNFLYKEKYNHLFYIFSHFDNITFKREVQNLIKKHKDEVIMDRQDyDCCFNLEGNVFNLCFCINEN-DKKYKCDNTRCY
HPC_PolZ             TLNYLYRNKHYLLLYILSNCNELVLKKKIQFIIETDYEKIKEEQNE--CCLTNPNNILKICLCVKHTgDGNERCDSNNCY

Pf3D7_1037000        CNVKQAMFFFYNP-TYNkvdYVFNTKLFYSHLLSHIRQTLSEYFNKIYDNQISCDNFIIYRKVKLGTYKGEMQGIRRKVS
LF_PolZ              CNIKQSMVFY-HA-EMN---YVFHTRPFYEHLSSQVQKVLLSIFIDVYENKIPLDNFIIYKQVKLGTYKGELPNQKRKVP
PHC_PolZ             CNFRQNYFFYVEN-NES---YVFESKLFYKQLSMKIENVLHFIFNKIYKNEISIDNFLIFKKVKLGTYVGELENLKRKVP
PHT_PolZ             CNFRQNYFFYVEN-NKG---YVFESKFFYKHLSMKIENVLHFIFNKIYKNEISLDNFLIFKKVKLGTYVGELENLKRKVP
HPC_PolZ             CNVKQATFLHYSGgNNNttyFIFNKKFFYSYLSLTIENVLSTYFNKIYNNTIPYNEFIIYKKVRLGTYKSELKNNKRKTP

Pf3D7_1037000        LPPQAIVARKIMRDFPNSIITYKEKVPYIFTKKLKDDKIYSSVSHPHFIRGIYRSFRFDCGSTEQLE--ISEDNCNNDSE
LF_PolZ              LPHQAIAAKRMYQLFENCLILYKDKIPYVVSTKLKKEKIYESVSHPFFIKGLYKSCKKYFENYSALEksTEEDEDEDESE
PHC_PolZ             LPPQAIVAKKLQHIFKNTMISYKERIPFVVVKKLKEEKMYTAVCSPFFLKGMFKSC------------------------
PHT_PolZ             LPPQAIVAKKMQHIFKNTMISYKERIPFVVVKKLKEEKMYTAVCSPFFLKGMFKSCSNVFKNYHQIFqhLLEIKNNNQIQ
HPC_PolZ             LPLQAIVAKKLLKTYSNVIISHKEKIPFIITQKLKGDKLYDSVSHPFFIKGIHKTKKEELKN------------------

Pf3D7_1037000        KNGDiEKNDNIEKND   -NIEKIGDIEKIGDIKKIGDIEKNENIEKNENIEKNDN[125]KFNWNK-AKLEMFSKYYKN
LF_PolZ              KEKEfNKKENRKKNK[6]LNYEYYIENLVLPPLKRMLDLLPFSTLNLEKIAHMARR     KYNS----NYYQMSFFRNL
PHC_PolZ             ---------------   --------------------------------------     -------------------
PHT_PolZ             IQNQ-NQEKKEKKNE[6]INYEYYISNATIPPLKRMLDLLPFYPLDLEKIFYNTRR     GGNDLKtQSIEETSNIINK
HPC_PolZ             ---------------   INFNYYIENLIIQPVKRMLDLLPMCSINLEKIFFKTKK     QYNLNK-GKIDIFSAYYGS

Pf3D7_1037000        MDNKLLK--NNNVTNKGINLMNILNEDKINNK[49]KIMNSYVEINKSQNMLKKLNKICLMCANSEMEALACNYSVHCKI
LF_PolZ              QNEHSLNkeSNKEIRQEKYVTELLEEEENQQT    CIIKSYIKINELQNKMKNVNRKCLECTNSEVKALACQYSIHCPI
PHC_PolZ             --------------------------------    ----------------------------------------------
PHT_PolZ             RKKIYINmeGNNEYEKRKEK----EKEKERDK    EIINSYILLSKYQNEMKKLNHICFSCAHSEIKALGCSYAIHCPV
HPC_PolZ             NTKKLLN--NNAKYNKKIKIKITTNNSKIENK    KIINNYIELNKSQNMLKRLNKICLACANTEMEAMACNYALHCKI

Pf3D7_1037000        FFKRINLQENILKNKDSINrls
LF_PolZ              YLKKLQLEEQILIQQEIVE---
PHC_PolZ             ----------------------
```

PHT_PolZ	YLKKLNLEEKISKCKEITSr--
HPC_PolZ	YFEKNLLVENISKNQKV-----

SNF2 Helicase Alignment

```
Pf3D7_0604600     [438]FLYNKKFINFIE-SIKNRKG-IyKKVLNKYLSAYLKNDFFNEINYLPLTWKPRKDYIKAGIFPFNLSKNNRKIES
LF_SNF2                --------------------------------------FFNEMNYLPLTWKPRKDYINSGVFPFYLLKKTKKIES
PHC_SNF2               FLYNWKFQQFIEyQIKVRKDeKiKKRTKRILSKCMDNDFFKKINYLPITWKPRKDNIESAIFPFYLSKKVKKKKT
PHT_SNF2               ---------------------------------------------------------------------------
HPC_SNF2          [332]FLYNEKIVHMLE-MEWSKKY-L-KCLMNKYLSTYLKNDFFQEMNYFPLTWKPRKDYIKSGIFPFYFAKNVKKINS

Pf3D7_0604600     FGRLSVNNNNYLCGCVFPLCTYQLIVRYFIYKLFHTCFIYQIPNFIFYFFRYYYRinNNINKHKMYNYFLKNKYINIFIN
LF_SNF2           FRKLSCYNNNYLSGSVFSLSSYPLIIRYFVSKFSHSFFIYQIPQTVLHFFSYFYP--TYNNKYRLHRYFLRDQYSSLLEK
PHC_SNF2          FRKWSIYNNEYLSGCVFNLCSYRLIFRYFISKFMDIYFIYEIPQTIFYFFRHFYK--KKYRKLNCYQYFLKDQYTNVLKD
PHT_SNF2          --------------------------------------------------------------------------------
HPC_SNF2          FKRLYVNNNNYLAGCVFNLCTYHLILHYFIYKNFETCFLY------------------NLN-------------------

Pf3D7_0604600     IYhSKYKQILDTIKEE[15]QKE[7]IKNLYLFDQLSNNTYIFSKQKIEKKIINHYYHKKIQKIKRQMNQEIKNICLNEI
LF_SNF2           EL-TNWEREEIQIQER[16]EKE   ILDQYHYEELCMNTYIFEKTELKKEIINHYFMKKECKIRKRSHKKIKEMCLDQI
PHC_SNF2          IM-AKYKSPIITLKEK    ELE   VLKSYDFDELCNSTFFLDKEQIRMEIIENYYMKQKNKIQKKIQKELKMCLKQI
PHT_SNF2          ----------------    ---   ------------------------------------------------------
HPC_SNF2          ----------------    ---   --------ELCNNNYIVSRKKLTKEIYKNYFEKEKHKLTKKFHKQMKELCLNEI

Pf3D7_0604600     KKKLPIRIQKVILTYQLQAIYFFFKKRGRILIADEMGLGKTLQAISIFYFYHLYPVLIITPASLKINWFSEIEKYLPAFD
LF_SNF2           KRKLPRRIRNVILPYQLETIYFFKKKGGRILVGDEMGLGKTLQSISIFSFFKLYPVLIVCPASLKTNWLCEIKKFVPFLD
PHC_SNF2          KKQLPLAIQKIILPYQLETIYFFKKRKGRILLGDEMGLGKTLQAISVFSFFQLFPVLIICPASLKINWLREIEKFVESLD
PHT_SNF2          --------------------------------------------------------------------------------
HPC_SNF2          KNKLPCKIKNVILPYQIETIYFFLKKKGRILIGDEMGLGKTLQAISIFSFYHLYPTLIVVPTSLKIHWLSEIEKYLPQFC

Pf3D7_0604600     PQNVLIINSSNDMPKCASSYKIIIVSFNIYKKLYNLLKEIQFHLIIVDESHFIRTVHYGNQSQLTRLLKKKIRKTKHVLF
LF_SNF2           PSKILRISSSNDVPNDLKKYKIIILSFHMFRHTFHFIRFIQFQLLVVDESHHIRSVHSGKESQLTNMMGLLTKQIKRVVF
PHC_SNF2          LSFILLVNSSNDLPKLHAHYKVIITSYEMFKRLYDVLKERTFRLLIVDESHYIRTVYCGKESKFTETIKELAKRIKNVLF
PHT_SNF2          ----------------------------MFKRLYDILKERTFRLLIVDESHHIRTVYCGKESKFTETIKELTKKIKNVLF
HPC_SNF2          ITQILVIHSSNDCPRLDARYKIIIVSFDIYKRLYNVLKQIEFKLVIVDETHHIRTVQYGKQSQLSKKLKNKIINSKYVIL

Pf3D7_0604600     LSGTPSINRPINIFHQIKYLINNKNIFPKNKIIFGEDYCKKYFYRGEKIYEENLRSWEFHYFLNKIVMIRRTINQVFQN-
LF_SNF2           LSGTPSVNRPINLFHQIYLLIQNDSIFCNTKFIFGEEFCKRSMSRGERKYEENLRAWEFSLFLKKTVLIRRHISSVLDSl
PHC_SNF2          LSGTPSVNRPINIFHQLKILINNKNIFPQNKYIFGEEFCKKICRRGEKIYEQNLRSWEFFMFLKKTVLIRRLLQQIFKE-
PHT_SNF2          LSGTPSVNRPINIFHQLKFLLNNKKIFPKNKYIFGEEFCKKICRRGEKIYEQNLRSWEFFMFLKKTVLIRRLLQQMFKE-
HPC_SNF2          LSGTASVNRPINIFHQIKYLINNNKLFFKNKYVFGEECCKKYIHRGEKIYEENLRSWEFSLFLKKIVMIRRNIKIIFKN-
```

```
Pf3D7_0604600      NFPSLKRFFVYLKNDICDN[11]HEN   SFINNSE[10]KCGYKMDEMYKMNNLDKNNN[11]DDQNEYKH[12]SKKP
LF_SNF2            QFLQLKRFFVFLPQEVKNE    ---   -------    --------------------    --------    ----
PHC_SNF2           NFPNLRRFFVPLSDPSMDT    ---   -------    --------------------    --------    ----
PHT_SNF2           NFPNLRRFFIPLSDPFMDK[11]TDE[6]ETLHTNL[10]NNNHLNNNHLNNNHLENNHL[10]TEIGTEIG[ 5]CKCI
HPC_SNF2           NFINLKRYFIFLPKD----    HDK   SSIHSSR    KILDKNESIEKNDNTDKHNT    DKYNTDKY[ 1]TDKY

Pf3D7_0604600      EQSKLNNSIIYKQERKHKNREILNQYFHIDIK   SKKEE    EGVSKIV    -NALQFIEKNFPNKKKIIFCY-HIM
LF_SNF2            -------MKEKENEVETNRREKLNKIYQVKL-[1]SKKEE    EGILKI-    KEGIEWIESHFPNEKKILLCY-HLV
PHC_SNF2           --------------------------------   -----    -------[ 4]KKEMKQIKIENKNVKKIILCNeHDL
PHT_SNF2           EEELLKLIKKKEQEQEQEQEQKQKQVHSPCIN[4]DKKKE[49]EKVQKIK[90]KKEIKQIKIENKNVKKIILCNeHDL
HPC_SNF2           NTDKHDNTDKHDNTDNTTNRQKLNDFFHVNIK   SKKEE    EGLSKIV    -NALEYMETYFPTKKKIIFCY-HLI

Pf3D7_0604600      VSK--CTEDELLKIIKQKK   EKENISIDYVSLNGCIPEKEK-IEKILYFQNNINCYYGIFTICSVSHGLDFSFCNLCF
LF_SNF2            ICK--CIEDELLKRIAKKK   EEDTSLLDYVVINGKVSEKHK-VERINYFKTNQTCTYAICTIGSVSHGFDFTFSNLCF
PHC_SNF2           FCDgdCRKVYFLKKSSNSL[5]ERDQGICN--VCNLDCSDLLKqIRRMKYFSMEEKINYFI------------KYNPL--
PHT_SNF2           FCDgdCRKFYFLKKSSNSL[5]ERDQGICN--ICNLDCSDLLKqIKRMKYFSMEEKINYFI------------KYNPL--
HPC_SNF2           VCK--CIEE----------   -----SIDYVVLNGSLNEKEK-LEKINYFKNTSNCYYGILTICSVTHGLDFSFCNLCF

Pf3D7_0604600      FMEFPVNFFHLQQCESRLFRKNQKHNTYVFYF--LLQKGLGSDHKTWNRFILCSNSTRSIIDGTNFINKDLIYDNI[467]
LF_SNF2            FFELPVNFFHLQQCESRIFRRNQTKDTYVFYF--LLRKGLGSDYNTWNRFLKCANATHSVVDGVNFDHSDL-----
PHC_SNF2           FLE------NINQLKKII---DSPKEGYIWQVdhIIPVYKGGGQASFENLQTLCTFCHQKKTKCDLQNKKEIKTRK[  3]
PHT_SNF2           FLE------NINQLKKII---NSPKEGHIWQ---------------------------------------------
HPC_SNF2           FIEFPIDFFHLQQCEARLFRRNQLYDTFVFYF--LLKNGLGSDEKTWKRFVLCAHSTRSTVDGTCYEGNDLFYEKF[410]
```

RING Finger Ligase Alignment

```
Pf3D7_0415800      [8]VQNNLNRARHSLTNTLSDMRLDE-SYLKSIRMNIKKRFDKLMNKIIPFET---IDENKRFVVIIEKKKN-YENFRCP
LF_Ligase             -----ESLKQSLLSQYEEQSEDP-DFIYNFRKHVKRTFELWIDRVVPLNR----LVDRRFIVLVENRKN-YDNVRCP
PHC_Ligase            IIEKTNEARNILVKQVNELKAED-ITLSVCRSYIQEKMKNFIDVCIPA----KIIKERRYIVIKDKHKH-YETFQCA
PHT_Ligase            -----------LVNQVNELKEED-ITLEVCRSYIQEKMKSFIDVCIPA----KVIKEKRYIVIKDKYKH-YEAFQCA
HPC_Ligase         [8]INNNMENIRNNVIKAFENLRQIDnIYVNNVRTEIQKKVNSLLYKCLPLSEdkKIINERRFIVIIEKDKNkYDNFRCP

Pf3D7_0415800      ICMLILFKPVKTKCGHIFCRECIEKVLLKFDYCPLCRNFIK-DKKLENVENSTLGSEYENIKIRCYKCKEITNIKNYEKH
LF_Ligase          ICMLVFYKPVITNCGHTFCHECLRKSVDKYACCPMCREPIApDSSLSEINAEVLGSEYSSIKIRCSVCRDVMTIKDYDPH
PHC_Ligase         ICTFLFYKPVLTPCGHIFCGDCLKRSLKNFKNCPMCRNDIT-EETIKDIPAEYLGEIYTQLVVRCPICRYKTTVGEYKGH
PHT_Ligase         ICTFLFYKPVLTPCGHIFCEDCLKRSLKTFRSCPMCRHEIR-EETVKKIPAEYLGEIYTQLVVRCPICRFKTTVGDYKRH
HPC_Ligase         ICMLILFQPVQTQCKHIYCKECIEATLKKYNHCPLCREVTS-NQTLTDLPISYFGREYTNIKIRCYKCKQITTIEKYEQH

Pf3D7_0415800      IINHI[26]IQKKKKNSN[4]ISNNNYIHNIYHHINPYNFDSYFNKKFQIIYMKEFFNLLKEhNINTHIDNFYLLYAQNV
LF_Ligase          LVNKC    YSNRMKYRE[4]SIDNDITNKTKTNAHLKKLKNVFGKQFDINQMKELKRLLKK-STFDHIRHFSLVYAKSM
PHC_Ligase         L----    ---------   ------------------------------------------------------------
PHT_Ligase         L----    ---------   ------------------------------------------------------------
HPC_Ligase         LKNHI[ 1]YYGNKNYKN   NYSNYNSYLERTVKNLKNIKTLFNKKIRINEMNTFLLYIENiSIITDIYSIQLVYGRKV

Pf3D7_0415800      LYDFKH[607]
LF_Ligase          KEEHIV[389]
PHC_Ligase         ------
PHT_Ligase         ------
HPC_Ligase         FNEAIY
```

Supplemental Figure 1: Amino acid sequence alignments for orthologues of the four components of the translesion polymerases described in the text.

Sequences for *P. falciparum* were obtained from the EuPathDB database while the additional orthologous sequences were obtained from the fragmented genome assembly of *Parahaemoproteus tartakovskyi* (Bensch *et al.,* 2016), the transcriptome datasets of *Parahaemoproteuscoatney* and *L. fringillinarum* (Galen *et al.*, 2019) and sequence data of a *Hepatocystis* parasite that was mined from the transcriptome of a Ugandan red colobus monkey (Simons *et al.*, 2019) using the ContamFinder pipeline (Borner *et al.*,

2017). Alignments were generated using the Constraint-based Multiple Alignment Tool (COBALT; Papadopoulos and Agarwala, 2007) available through the National Library of Medicine, National Center for Biotechnology Information website.

Alignments are displayed in the "compact" format to reduce space. Unaligned regions are displayed as [X] where X denotes the number of residues for a sequence in the unaligned range.

References

Bensch, S., B. Canback, J. D. DeBarry, T. Johansson, O. Hellgren, J. C. Kissinger, V. Palinauskas, E. Videvall, and G. Valkiunas. 2016. The Genome of *Haemoproteus tartakovskyi* and Its Relationship to Human Malaria Parasites. *Genome Biol. Evol.* 8:1361-1373.

Borner, J. and T. Burmester. 2017. Parasite infection of public databases: a data mining approach to identify apicomplexan contaminations in animal genome and transcriptome assemblies. *BMC Genomics* 18:100.

Galen, S. C., J. Borner, J. L. Williamson, C. C. Witt, and S. L. Perkins. 2019. Metatranscriptomics yields new genomic resources and sensitive detection of infections for diverse blood parasites. *Mol. Ecol. Resour.* doi:10.1111/1755-0998.13091.

Papadopoulos J.S. and R. Agarwala. 2007. COBALT: constraint-based alignment tool for multiple protein sequences. *Bioinformatics*. 23(9):1073-9.

Simons, N. D., G. N. Eick, M. J. Ruiz-Lopez, D. Hyeroba, P. A. Omeja, G. Weny, H. Zheng, A. Shankar, S. D. W. Frost, J. H. Jones, C. A. Chapman, W. M. Switzer, T. L. Goldberg, K. N. Sterner, and N. Ting. 2019. Genome-Wide Patterns of Gene Expression in a Wild Primate Indicate Species-Specific Mechanisms Associated with Tolerance to Natural Simian Immunodeficiency Virus Infection. *Genome Biol. Evol.* 11:1630-1643.
